# Ecological Succession of Polymicrobial Communities in the Cystic Fibrosis Airways

**DOI:** 10.1128/mSystems.00809-20

**Published:** 2020-12-01

**Authors:** Rutvij A. Khanolkar, Shawn T. Clark, Pauline W. Wang, David M. Hwang, Yvonne C. W. Yau, Valerie J. Waters, David S. Guttman

**Affiliations:** a Department of Cell and Systems Biology, University of Toronto, Toronto, Ontario, Canada; b Department of Laboratory Medicine and Pathobiology, University of Toronto, Toronto, Ontario, Canada; c Centre for the Analysis of Genome Evolution and Function, University of Toronto, Toronto, Ontario, Canada; d Laboratory Medicine and Molecular Diagnostics, Sunnybrook Health Sciences Centre, Toronto, Ontario, Canada; e Department of Pediatric Laboratory Medicine, Division of Microbiology, The Hospital for Sick Children, Toronto, Ontario, Canada; University of Southampton

**Keywords:** *Pseudomonas aeruginosa*, anaerobic bacteria, cystic fibrosis, ecological succession, microbial ecology, microbiome, polymicrobial infections, pulmonary exacerbations

## Abstract

Antimicrobial therapies against cystic fibrosis (CF) lung infections are largely aimed at the traditional, well-studied CF pathogens such as Pseudomonas aeruginosa and Burkholderia cepacia complex, despite the fact that the CF lung harbors a complex and dynamic polymicrobial community. A clinical focus on the dominant pathogens ignores potentially important community-level interactions in disease pathology, perhaps explaining why these treatments are often less effective than predicted based on *in vitro* testing. A better understanding of the ecological dynamics of this ecosystem may enable clinicians to harness these interactions and thereby improve treatment outcomes. Like all ecosystems, the CF lung microbial community develops through a series of stages, each of which may present with distinct microbial communities that generate unique host-microbe and microbe-microbe interactions, metabolic profiles, and clinical phenotypes. While insightful models have been developed to explain some of these stages and interactions, there is no unifying model to describe how these infections develop and persist. Here, we review current perspectives on the ecology of the CF airway and present the CF Ecological Succession (CFES) model that aims to capture the spatial and temporal complexity of CF lung infection, address current challenges in disease management, and inform the development of ecologically driven therapeutic strategies.

## Abstract (precis)

Antimicrobial therapies against cystic fibrosis (CF) lung infections are largely aimed at the traditional, well-studied CF pathogens such as Pseudomonas aeruginosa and Burkholderia cepacia complex, despite the fact that the CF lung harbors a complex and dynamic polymicrobial community. A clinical focus on the dominant pathogens ignores potentially important community-level interactions in disease pathology, perhaps explaining why these treatments are often less effective than predicted based on *in vitro* testing.

## INTRODUCTION

Cystic fibrosis (CF) is a lethal autosomal recessive genetic disease caused by mutation of the cystic fibrosis transmembrane conductance regulator (CFTR) gene that encodes a transepithelial chloride transporter (1). CF primarily occurs in the Caucasian population, with an incidence rate of 1 in 2,500 newborns and an estimated 70,000 individuals affected worldwide (2). Currently, two-thirds of individuals are diagnosed during the first year of life due to widespread newborn screening (3).

Although CF is a multiorgan disease, respiratory complications are a significant source of morbidity and mortality (2). Chloride imbalances caused by CFTR mutation lead to changes in normal lung physiology that promote the accumulation of viscous dehydrated mucus, decreased mucociliary clearance, inflammation, and an altered immune response. These factors ultimately contribute to the establishment of chronic infections and an irreversible decline in lung function secondary to bronchiectasis (4). As a part of progressive respiratory disease, patients also experience cycles of acute intermittent aggravations of symptoms called pulmonary exacerbations (PEs) which may be characterized by a drop in lung function (measured as forced expiratory volume in 1 s [FEV_1_]), shortness of breath, chest pain, and increased cough and sputum production (5, 6). Approximately 25% of patients fail to recover baseline lung function following PEs (7). The exact cause of PEs remains unclear, making effective advanced prediction and prevention difficult.

Current approaches in the treatment of CF lung infection center around the use of broad-spectrum antimicrobials and airway clearance techniques to reduce the microbial load and clear mucus obstruction in an attempt to improve lung function (8). These approaches predominantly focus on the detection of “traditional CF pathogens” in respiratory cultures, including Pseudomonas aeruginosa, Staphylococcus aureus, Haemophilus influenzae, and Burkholderia cepacia complex (BCC) species such as Burkholderia multivorans and Burkholderia cenocepacia, among others (9). In addition to traditional pathogens, other bacteria, viruses, and fungi are typically identified concurrently in CF sputum specimens. Contrasting hypotheses have been proposed to describe their role in lung disease, including being involved in complex infectious polymicrobial networks or representing upper respiratory contaminants introduced during expectoration. However, it is typically recognized that CF airway communities are heterogeneous and differ in composition both spatially and temporally (10–16). Despite this understanding, the traditional pathogens are typically assumed to be the primary drivers of CF lung disease. The reliance on this assumption may help explain why antimicrobial therapies fail all too frequently (17, 18). Characterizing CF lung infections as ecologically homogeneous and driven by a few dominant species minimizes the potential importance of complex and dynamic ecological interactions.

Despite the potential diversity of the CF microbiome, culture-based microbiological approaches often report negative findings when traditional pathogens are not isolated (19). This may occur for multiple reasons, including the use of culture conditions specific to the traditional pathogens. Most microbial species are difficult to culture (19), and their clinical significance is frequently unclear despite being relatively common in CF airways and relatively rare among healthy individuals. Finally, since culture-dependent analyses are predicated on the notion that a one or a small number of pathogens are responsible for infection, they may be a poor clinical tool for CF respiratory specimens (19–21).

More recently, culture-independent methods have been applied to better characterize the CF lung microbiota. These approaches identify microorganisms in respiratory specimens in the absence of *in vitro* culture by sequencing PCR amplicons derived from either a hypervariable region of the bacterial 16S rRNA or fungal ITS genes (i.e., microbiome sequencing) or by shotgun sequencing all nucleic acids present (i.e., metagenomic sequencing). Culture-independent methods have allowed for greater in-depth examinations of microbial communities, allowing for the detection of many bacteria using 16S rRNA gene sequencing that were not previously identifiable using culture-dependent methods (22). However, these methods are still limited since 16S rRNA gene-based analyses typically do not permit species-level identification of taxa, produce count-based data that only measures the relative abundance (as opposed to the absolute abundance) of taxa, can potentially be confounded by DNA from environmental or nonviable sources, and can only infer host-microbe or microbe-microbe interactions and the metabolic potential of individual taxa or the community as a whole (12, 23–26). Reconciling the limitations of culture-dependent and -independent methods is difficult and will require a better understanding of whether the existence of polymicrobial CF communities also means that multiple pathogens contribute to the pathology of CF infections.

Microbes regularly influence their environment both directly (e.g., through the production of biofilm or metabolites that elicit a host response) and indirectly (e.g., through their interaction with other microbes); consequently, there is every reason to believe that the specific composition of the CF lung microbial community may have a strong influence on patient clinical status (8). Recent findings suggest that ecological interconnectedness of microbial communities may be more effective in identifying specific taxa associated with changes in clinical states than simple correlations of relative or absolute taxa abundance (27, 28). However, the heterogeneity in the CF lung has made it difficult to accurately describe the spatial and temporal changes that are occurring. While recent studies have greatly improved our understanding of the range of microbes that reside within the diseased lung, there is still a strong focus on traditional respiratory pathogens, with the potential role of other, lower-abundance taxa remaining relatively undescribed. We (and others) (8, 28–33) argue that a more nuanced understanding of the dynamic ecology of the CF microbiota may strengthen our understanding of disease development and the role of microbes in PEs. We propose here that concepts of ecological succession and their framework for understanding the development of complex biological communities and how these communities respond to environmental perturbations may be useful. We hope that this ecological approach will permit the development of improved therapies in CF that may limit the selection of antimicrobial-resistant organisms.

## ECOLOGICAL MODELS OF CF INFECTIONS

The first studies to discuss the CF airway as a dynamic ecological system were by Harrison in 2007 and by van der Gast et al. in 2011 (16, 29). The minireview by Harrison explicitly focused on understanding the CF lung as a community, with coinfection, microbial interactions (synergism and antagonism), and evolution playing potentially significant roles in shaping the lung ecosystem (29). van der Gast et al. studied bacterial communities in CF sputum using Sanger-based sequencing of the whole 16S rRNA locus and was able to partition the community into core and transient groups by applying community ecology metrics (16). However, it was not until several years later that more general ecological models of the CF airways were described.

### Climax-Attack Model.

One of the most well-known ecological models of the CF airways is the Climax-Attack Model (CAM). Conrad et al. postulated in 2013 the existence of two broad functional states: a virulent, transient, attack state associated with PEs, and a stable, persistent climax community (8). Since these states are functional, they need not differ taxonomically, which is consistent with studies that show that the composition of the CF lung microbiome can remain largely unchanged between clinically stable and exacerbated states (34–40). Instead of compositional differences, each state is defined by differences in metabolic function, gene expression, or interactions between microbial species. In this model, the attack community includes anaerobic genera such as *Prevotella*, *Veillonella*, *Fusobacterium*, and *Streptococcus*, as well as eukaryotic viruses (8, 28). In concert with the host immune system and hyperinflammatory phenotype of CF airway epithelial cells, the attack community is thought to participate in airway remodeling that is characterized by progressive bronchiectasis, atelectasis, fibrosis, and vascular changes which negatively impact lung function (41, 42).

Airway remodeling promotes the colonization of climax species such as *Pseudomonas* spp., *Staphylococcus* spp., Stenotrophomonas maltophilia, and *Achromobacter* spp., which have a disproportionately large effect on the ecosystem relative to their abundance and which can be difficult to eradicate (8). The CAM model is supported by a recent study that measured microbial community composition and metabolic function in patients both pre- and postantibiotic treatment that found evidence of alternate climax and attack functional states (43). One alternative explanation for the stability of the microbial community during exacerbations is that PEs are associated with intrapulmonary spread of infection rather than changes in functionality or composition (36).

To test the CAM framework, Quinn et al. in 2015 developed the *in vitro* Winogradsky (WinCF) model to study how the physiology of the CF lung contributes to PEs (44). They created Winogradsky columns from narrow-capillary tubes filled with artificial sputum medium to simulate the biogeochemical gradients within CF bronchioles. After inoculating columns with CF patient sputum expectorated during PEs, they noted changes in pH, gas production, and community composition. Pretreatment communities (the first was PE treated with intravenous antibiotics, and the second with oral antibiotics) included abundant *Lactobacillales* and obligate anaerobes such as *Prevotella* and *Veillonella*, which produced large amounts of gas. Posttreatment communities were *Pseudomonas*-dominated and had a lower relative abundance of anaerobes. Similar to the CAM, two distinct functional states driven by changes in oxygen, pH, and metabolism were identified: one associated with aerobic metabolism and higher pH and a second associated with fermentative metabolism, lower pH, and potentially PEs. The former includes members of the climax community, including *P. aeruginosa* and S. maltophilia, while the latter is similar to an attack community (45, 46). A subsequent ecological network analysis found that the attack community correlated with lower pH, anaerobic genera, and fermentation, while the climax community was associated with a pH increase, and ammonia production via deamination (28).

### Island Biogeography Model.

Whiteson et al. developed the Island Biogeography Model (IBM) that draws parallels from concepts of island biogeography to describe the colonization of the CF lung. This model treats different human organs and tissues as “islands.” each with a unique microbiota. Similar to islands, structures of the human body experience migration of microorganisms from one environment or compartment to others, with the rate and type of migration dependent on the dispersal rate and ease of mobility between compartments (47–49). In this model, the source population has the greatest diversity and contributes to sink populations via emigration. In CF, the upper respiratory tract is typically assumed to be the source population or reservoir, but colonization also occurs from other “islands” such as the gastrointestinal tract. The source-sink relationship described in the IBM is supported by studies that have found that by 2 years of age, the lung microbiome of CF infants shifts toward a community dominated by organisms found in the oral cavity (50), and by bronchoscopy studies of healthy individuals, which report pulmonary microbiota with similar composition to that of the oropharynx (51–53).

The richness of an island’s species is based not only on the rate of colonization, but also on the replication rate of the resident taxa and local extinction rate. While the CF lung is a chronic inflammatory environment, some lung-specific immune responses are impaired, including dysfunctional alveolar macrophages and autophagy (54–56), reducing the rate of immune-mediated clearance (i.e., extinction). Further, the CF lung is a nutrient-rich environment, which should theoretically support higher replication rates and carrying capacities; however, recent studies have found reduced growth rates among many CF bacterial isolates (57, 58).

The composition of the CF lung community may also be indirectly influenced by physically separated species via the elicitation of systemic immune responses or production of metabolites that can be translocated into the lungs (59). For example, anaerobes of the oropharyngeal flora have been found to modulate *P. aeruginosa* virulence factor expression in the lower airways (60–62). In contrast to the CAM, which describes the existence of two functional states that are separated temporally, the IBM can explain spatial heterogeneity within the CF lung. A similar model was proposed by Boutin and Dalpke (63) and incorporated concepts derived from neutral theory. With this theoretical approach, these authors suggested that over time, disruption to the balance of migration and elimination among anatomic sites early in life is the main determinant of the lung microbiome composition rather than local selective processes (63). They also speculated that microenvironmental factors affect the interplay of immigrating community which favors the outgrowth of certain bacterial species in later disease stages.

### Limitations of current ecological models.

Although models such as CAM and IBM provide insight into critically important aspects of CF lung ecology, they focus on distinct facets of pulmonary infection and do not provide a unifying framework of the infectious cycle over time. For example, the IBM describes the mechanism by which microbes colonize and persist in the lung, but does not provide insight into the frequently observed spatial structure or metabolic diversity of the microbial communities in the upper and lower airways (64–69). It is unknown whether interlobe variability in community composition is driven more by stochastic variation in colonization success or local (deterministic) selection pressures.

One important aspect of CF lung ecology that has received surprisingly little focus in current ecological models is the temporal perspective of ecological succession. CF microbial communities, like all ecosystems, show not only significant spatial diversity, but also temporal variation. While the CAM provides a well-supported framework based on two functional states, it does not specifically address the dynamics of CF lung infections over the life-history of a CF patient (8). Here, we aim to describe the temporal succession of the CF lung microbial communities and their relation to clinical disease phenotype.

## CYSTIC FIBROSIS ECOLOGICAL SUCCESSION MODEL

**FIG 1:**
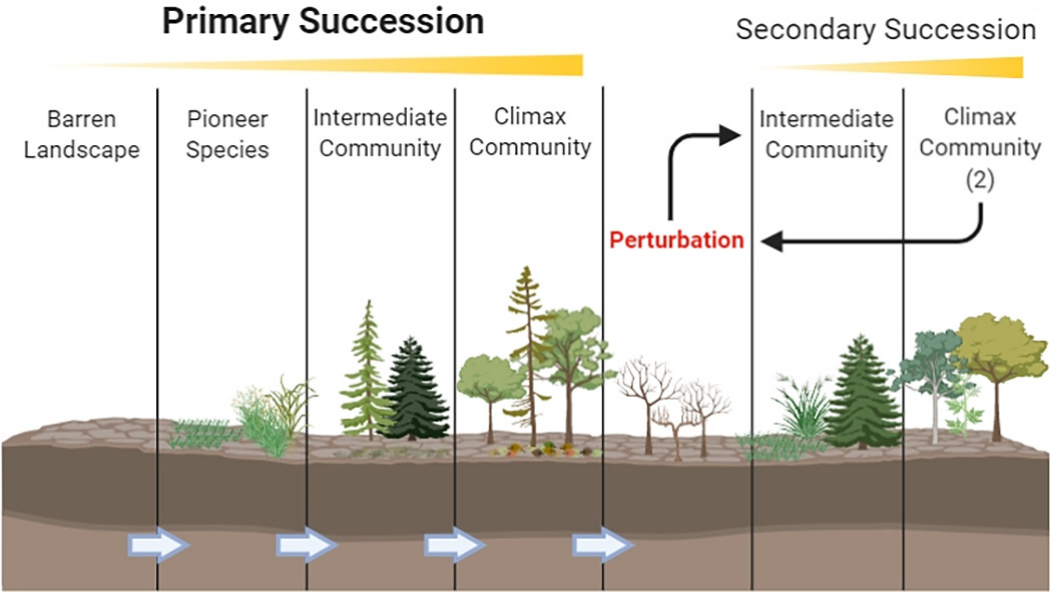
Model of ecological succession describing the stages of primary succession (left) that occurs through pioneer species which colonize an initially uninhabited landscape from an environmental reservoir secondary succession (right) that occurs through locally resident species following a perturbation that disrupts the normal steady state and results in the formal of a stable, but different climax community. As the perturbations, however minor, are constantly occurring, even a relatively “steady-state” community is constantly in a dynamic state of flux, and moving toward a desired equilibrium, which may never be truly reached. The image was created with BioRender.

The principles of ecological succession describe an initially barren environment that is colonized by pioneer species from an external environmental reservoir that can survive in a landscape that is inhospitable to other species (33, 70). Pioneer species can remodel the ecosystem over time in ways that allow for intermediate species to colonize. Finally, a stable-state climax community is formed—characterized by long-term persistence without significant change. This process is known as primary succession (70) (Fig. 1). The climax community is often disrupted by transient ecological perturbations. While the resilience of the climax community typically results in a return to the original steady-state, rare, more severe perturbations can permanently disrupt the community steady-state, resulting in a period of ecological shift and the establishment of a new, but different steady-state (71). This type of succession occurs through species that were local residents of the previous ecological community rather than species that are colonizing from other environments and is termed secondary succession (Fig. 1).

The CF lung ecosystem, like all ecosystems, follows a pattern of ecological succession. The ecosystem begins as a sterile environment at birth and is initially colonized by pioneer species, following a period of succession until the first stable climax community forms. Minor ecological perturbations, such as the introduction of a new species, resources, or stresses from an external environment are frequent but have little impact on the steady state of the climax community. In contrast, major perturbations can greatly impact the stability and composition of the ecosystem, leading to secondary succession and the establishment of a new steady-state climax community. Ecological theory also predicts that the greater the frequency and intensity of perturbations, the less predictable the path and endpoint of succession (72).

**FIG 2:**
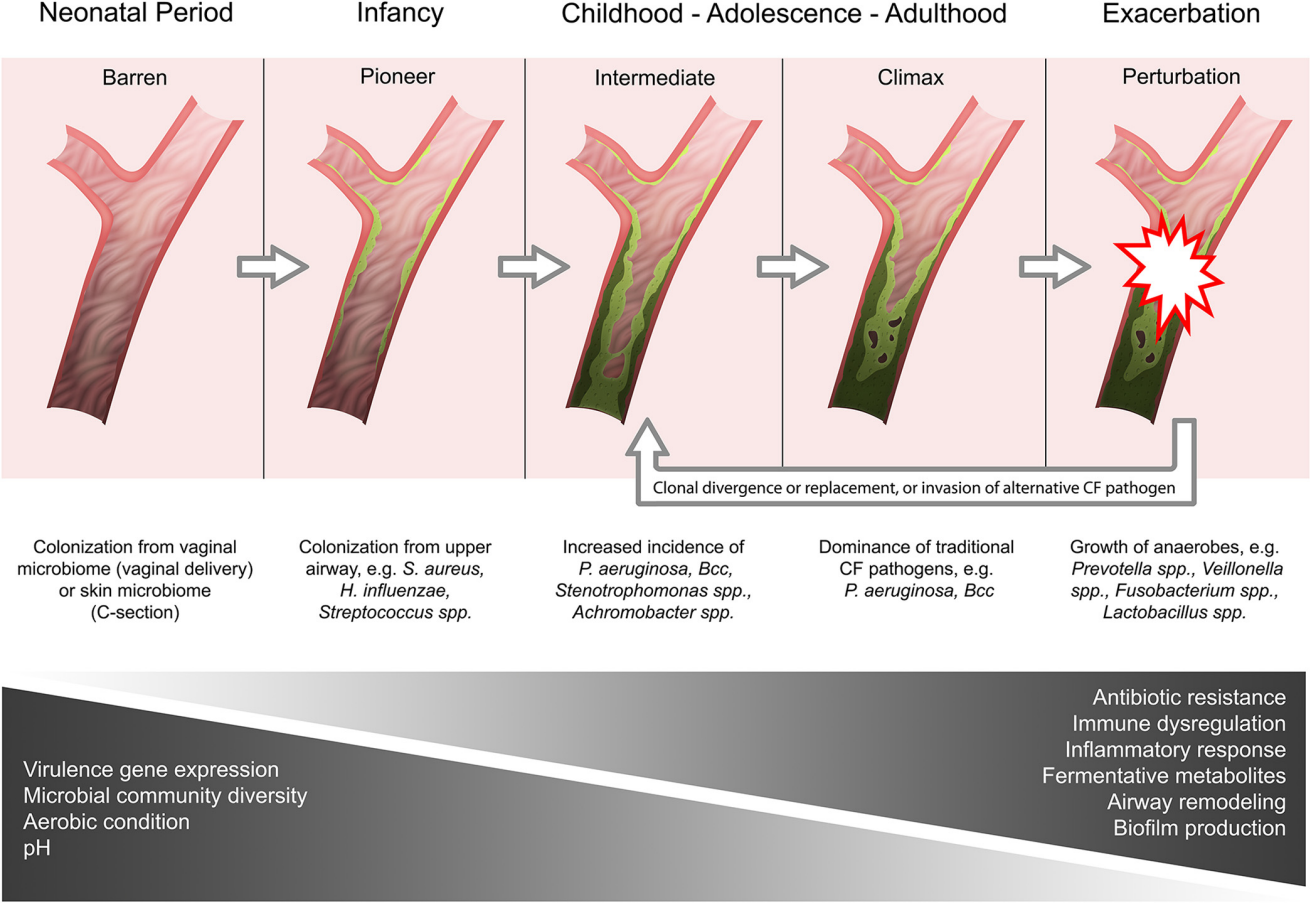
CFES model showing progressive changes to the lungs over time and disease development. Each stage of development corresponds to an analogous stage of succession shown in Fig. 1, along with the microbial taxa frequently observed in the lung at that stage. The two gradients below the lung diagrams illustrate traits that progressively change during the stages of succession, with light gray and dark gray indicating low and high levels, respectively. (Copyright Maggie Middleton.)

The Cystic Fibrosis Ecological Succession (CFES) model (Fig. 2) aims to describe the life-history of CF lung infections, beginning with initial colonization at birth and following the stages of progressive CF lung disease and deterioration, which eventually lead to premature death. We draw insights from the CAM and IBM models in describing various elements of CF lung ecology, including PEs, spatial heterogeneity within the CF microbiota, and the colonization and diversification of bacteria.

### Pioneer colonization.

Culture-dependent methods have long suggested that lungs are sterile at birth, and colonization may relate to the mode of delivery (73, 74). While vaginal delivery results in oral colonization by vaginal and gut microbes, caesarean section deliveries result in oral colonization by microbes predominantly found on the skin and in the hospital environment and is associated with reduced oral microbiota diversity (74–76).

The upper-airway, including the oral, nasal, and pharyngeal cavities, is believed to be the source population for lower-airway colonization (50, 77, 78); consequently, differences in CF versus non-CF upper airway microbes may result in different pioneer species during the early colonization of the lower airways (33). As early as the first few months of life, significant differences are observed between the nasopharyngeal microbiotas of CF infants and healthy controls, with a higher relative abundance of *Staphylococcus*, *Streptococcus*, and *Pseudomonas* spp. observed in CF infants (79). The early pattern of succession was also found to be different, with CF infants seeing a shift from early *S. aureus* and H. influenzae dominance toward *Streptococcus* and *Moraxella* spp. by 3 months of age, while the microbiota of healthy infants maintains a steady dominance of *Moraxella* spp., *Corynebacterium* spp., and H. influenzae (79). While the driver of the differences between healthy and CF individuals is unknown, inflammation of the CF airways in the first few months of life is detectable despite the absence of clinically diagnosed infection (80, 81). This inflammation may be due to undiagnosed infections, altered immune function, or decreased mucociliary clearance (80–85).

Muhlebach et al. (50) used both cross-sectional and longitudinal sampling via bronchoalveolar lavage (BAL) to characterize the lower airway of CF patients ranging from less than 1 year old through 5 years old and found that young CF patients had few microbes in their lower airway, and the samples were largely indistinguishable from background contamination. In keeping with the IBM, in the first year of life, infant lungs had been colonized by a community dominated by oral and upper airway microbes (50). A similar trend was observed in a longitudinal study of CF infants (<1 year of age) over a mean duration of 14-months where *Streptococcus* and *Haemophilus* spp. dominated communities from throat swabs, with *Staphylococcus* and *Pseudomonas* spp. being rarely detected (20).

In contrast, Jorth et al. (86) reported that compositions in the early lung microbiota differed in 22 children and young adults with CF ranging in ages from 6 to 21 years old. In BAL samples, bacterial communities consisting of *Streptococcus*, *Prevotella*, and *Veillonella* among others, did not establish prior to traditional CF pathogens. Analysis of paired BAL samples and analyte negative reagent controls (standard reagents used during sequencing experiments in the absence of clinical samples) indicated that nonconventional taxa detected in low-biomass BALs were likely contaminants from processing reagents and did not reflect true microbial communities (86). In samples where typical oropharyngeal genera were found at an increased relative abundance compared to controls, they were associated with and vastly outnumbered by traditional CF pathogens (86). These findings contradict existing theories and suggest that the pioneer species may in fact be traditional CF pathogens and that anaerobic or environmental taxa detected earlier on through 16S rRNA gene sequencing may simply be nonviable or transient contaminants from the upper airways.

### Primary succession and the intermediate community.

Early colonization events are followed by stages of succession. Infections in infants and young children with CF typically harbor *S. aureus* and H. influenzae, with rates of infection by *P. aeruginosa*, S. maltophilia, *Achromobacter* spp., and BCC. becoming more common through adolescence and young adulthood (87). By 3 years of age, serological evidence of intermittent *P. aeruginosa* infection is found in 95% of children (88). The sources of traditional CF pathogens such as *P. aeruginosa* and BCC are believed to be environmental reservoirs since most of these species are commonly found in soil, although interpatient horizontal transmission is also well described (89, 90). While the sources of these infections are not entirely clear, the consequences are, since colonization by *P. aeruginosa* and BCC are strongly associated with poor prognosis, frequent PEs, and decline in lung function (91, 92).

In general, the underlying drivers of succession in this ecosystem are unknown, although it has been shown that the presence of some microbes can facilitate or hinder the colonization by others. For example, *S. aureus* infection has been found to predispose the lungs to future *P. aeruginosa* infection (65, 93). Interactions between *P. aeruginosa* and *S. aureus* in CF are well-recognized and recently reviewed by Hotterbeekx et al. (94). These interactions are often metabolically driven. For example, *P. aeruginosa* preferentially utilizes *S. aureus*-produced lactate, while generating metabolites that can, in turn, reduce *S. aureus* viability. These observations are supported by an inverse correlation between the prevalence of *P. aeruginosa* and *S. aureus* (28, 95–98) and *in vitro* studies showing that *P. aeruginosa* competitively excludes *S. aureus* in coculture (99–103). Other inhibitory mechanisms such as the sequestration of iron and production of antimicrobials also contribute (99, 104–106). These data are in agreement with the CAM, which argues for significant remodeling of the airways in early life by attack communities prior to the establishment of chronic CF infection by traditional pathogens (8).

Changes to the CF lung microenvironment as the disease progresses are believed to cause drastic shifts in community structure. The lung is a heterogeneous and compartmentalized organ with variable oxygen and chemical composition due to the continued accumulation of mucus and diseased tissues and microaspiration events. This can introduce variation in local conditions within the smaller airways. It is possible that a spatial component of succession events exists, the extent of which may be experienced differently across communities in individual lung compartments. As seen in the WinCF model, areas of hypoxia and variable acidity may create favorable niches for anaerobic bacteria that require alternate-electron acceptors for growth (9, 107). This may contribute to a temporary period in which microbial diversity increases and peaks during adolescence before significant remodeling of the lung occurs through attack communities (8, 108, 109). It has been proposed that the reduced pH associated with anaerobic fermentation may trigger 2,3-butanedione biosynthesis by *Streptococcus* spp. to avoid lethal acidification. These fermentation by-products may then be metabolized by *P. aeruginosa* to produce compounds such as phenazines (59). This may contribute to the success of *P. aeruginosa* in anoxic regions of the CF lung as phenazines can be used to relieve redox stress, in addition to other anaerobic respiratory pathways (31, 110–112). The production of 2,3-butanedione by anaerobic species can enhance *P. aeruginosa* biofilm formation and antibiotic tolerance and contributes to airway inflammation (62, 113, 114). Anaerobic conditions have also been associated with the formation of *P. aeruginosa* macrocolonies, which provides greater defense against leukocytes (107, 115).

Interspecies interactions, particularly those between anaerobes and traditional CF pathogens, may also play a role at this stage of infection. Examples of these associations include the production of antibiotic degrading enzymes by some anaerobes which provide traditional CF pathogens with defense against β-lactam antibiotics, and the production of metabolic by-products such as short-chain fatty acids (SCFAs) that can be produced by the metabolic degradation of airway mucins (116). SCFAs can drive powerful proinflammatory responses, marked by the release of cytokines in the bronchial epithelium, and are associated with neutrophil recruitment into the lungs (117). These factors may contribute to the increased relative abundance of anaerobes, metabolic fermentation, and community (alpha) diversity that is observed during PEs compared to baseline (27, 44, 118, 119). Some studies have suggested that changes in the abundance of obligate anaerobes such as *Veillonella*, *Porphyromonas*, and *Prevotella* may be potential predictors of PE onset (120, 121). However, there has been no reliable indication of whether anaerobic bacteria actually drive the onset of PEs or whether their growth is simply favored during the physiological changes that occur during exacerbations.

While many mechanistic studies suggest a role for anaerobes as participants or facilitators of CF lung disease, these appear discordant with clinical observations, as general anaerobe abundance and community (alpha) diversity often correlate with milder disease, greater lung function and body mass index, and decreased pancreatic insufficiency (16, 122). The relative abundance of *Prevotella* spp. in particular has been associated with increased FEV1, and lower inflammation and C-reactive protein levels compared to infection with *P. aeruginosa* and other traditional pathogens (123). The relative abundance of anaerobic genera and overall microbial community diversity has been shown to decline with disease progression and be inversely related to increases in the abundance of traditional CF pathogens (21, 119, 124). However, some studies have shown that FEV_1_ did not differ significantly between patients with and without obligate anaerobes and that their prevalence did not correlate with the presence of *P. aeruginosa*, as suggested by previous studies (118, 125). Since less-severe PEs tend to be treated with antibiotic regimens that have less anti-anaerobic activity, it has been suggested that differences between regimens may be a confounding factor that contribute to these contradictory findings (27). This conflicting information makes it difficult to elucidate the role of anaerobic organisms in CF lung disease. Correlations between anaerobic diversity and milder lung disease may simply be explained by greater microbial community diversity and anaerobes being more likely to be present in high-diversity communities. In CF patients older than 25 years of age, an increased anaerobe abundance has been observed, which may suggest that a “survivorship effect” exists, since a higher rate of anaerobes and greater community diversity correlate with a milder CF disease phenotype (25).

The role of anaerobes in CF lung disease progression may also be dependent on the specific composition of their associated microbiome, including the identity, relative abundances, and potential interactions with other members of the polymicrobial milieu. This has been noted particularly in *Prevotella* spp., which are some of the most common anaerobes found in the CF airways and constitute a large number of different species that may vary in their pathogenic potential (123, 126). Regardless of whether anaerobes carry a true pathogenic role themselves, are merely influential in the pathogenic behavior of other species, or are inert bystanders, there is mounting evidence to suggest that they are part of the “attack” community that is involved in significant remodeling of the airways (8).

### Climax community.

In a forest ecosystem, climax communities are characterized by the dominance of large trees that crowd the canopy, occlude sunlight, and use their deep root systems to monopolize water and nutrient access. This leads to the gradual elimination of previously abundant pioneer and intermediate species. The optimal conditions that promote the establishment of climax communities do not exist during the early stages of primary succession. Instead, the ecosystem is primed to support and sustain climax communities by the activities of pioneer and intermediate species. A notable example of this is the reconstitution of nutrient-poor soil by nitrogen-fixing pioneer species which is vital for the growth of larger plant species (70, 127, 128). Applying this concept to CF, while climax species such as *P. aeruginosa* and BCC may appear earlier in disease progression, they may be better suited to competing in the immunogenic, crowded, and heterogenous lung environment seen in later stages of CF disease after the airway microenvironment has been remodeled by the early pioneers and intermediate community (8, 12).

CF climax species also utilize multiple mechanisms to promote their own dominance over other genera in the lower airways, similar to the deep roots and tall stature of trees that enable them to outcompete intermediate species in the forest ecosystem (12). For example, ammonia production by some pathogens may stabilize lung pH and prevent the onset of PEs where conditions may favor anaerobic species (28). Both *P. aeruginosa* and BCC produce a variety of compounds such as 2-alkyl-4(1*H*)-quinolones and phenazines that can enhance their competitiveness by sequestering essential biomolecules such as iron or facilitate the use of alternate metabolic pathways under oxygen-limiting conditions, thereby preventing disruptions in community composition from constant colonization events (110, 129, 130).

### Perturbations.

Ecological disturbances or perturbations are any transient phenomena that result in a significant change to the structure of the community. Small perturbations typically have only short-term impacts followed by a return to the prior steady state, while large perturbations can push to community to an entirely new configuration. Perturbations can also be necessary for the establishment of specific climax communities. For instance, forest fires are a major perturbation that can decimate and remodel the landscape, but they also put nutrients back in the soil and open pinecones which will seed the next climax community.

In CF, the most frequently discussed perturbations are PEs, although perturbations can also be caused by antibiotic treatment, strong immune responses, other health-related phenomenon, and of course, lung transplantation. Determining the underlying cause(s) of PEs has been one of the most difficult problems in CF research. PEs not only pose acute, life-threatening health challenges to CF patients but also have long-term impacts since each PE can result in irreversible airway damage that accelerates lung disease progression (7, 131). Relative to healthy tissue, the fluid lining the CF airways shows a reduced pH even before infection, possibly due to impaired bicarbonate transport (132). This may facilitate the growth of acid-tolerant organisms such as *Lactobacillus*, *Prevotella*, *Veillonella*, *Streptococcus*, *Rothia*, and *Granulicatella* whose fermentative metabolism could sustain a low-pH environment (28). An increase in anaerobic fermentation can create a positive-feedback loop that favors the growth of anaerobic bacteria. Consequently, PEs have been associated with increases in both the relative and absolute abundance of anaerobic taxa (44, 118, 119) and a disruption in the composition and structure of climax communities (27, 28, 44, 133). These findings further support the existence of two functional states as described in the CAM, where low-pH environments favor acid-tolerant anaerobes, while environments with higher pH favor climax community pathogens (8, 28).

Ecological theory predicts that communities with greater diversity and richness of species are more resilient to perturbations because they have a greater functional capacity to adapt to environment change or resist invasive species (134–137). Diverse communities are less likely to experience intense disruptions and are therefore predicted to remain close to their desired steady state rather than constantly experiencing major succession events. Compared to healthy controls, analysis of both expectorated sputum and BAL fluid from CF patients has shown that lung microbial diversity decreases with age, often resulting in the establishment of a dominant taxa (i.e., found at >50% relative abundance) (12, 138). This may explain why PEs also become more common with age, as the ability of the microbiota to resist perturbations decreases with the decline in community richness (139).

### Secondary succession.

Communities will begin to return to a steady state following any nonterminal perturbation. Strains and species that survive a perturbation will expand to fill vacant niches, with the surviving diversity largely shaped by the selective pressures imposed in the prior perturbation, coupled with both local adaptation and stochastic events (i.e., genetic drift) (140–142). Unlike more well-studied macroecosystems (e.g., forests), postperturbation microbiomes may show less turnover in species composition but instead see a substantial turnover in clonal lineages. For example, following the antimicrobial treatment of PEs, a microbial community may consist of the same traditional CF pathogens as seen in the prior climax community but now be dominated by derived clonal lineages that have higher levels of antibiotic resistance (107, 142–145). The lack of a stable and resilient climax community may provide an opportunity for other species to colonize from extrapulmonary sites. However, the now significantly remodeled CF airways may be inhospitable to many organisms, which may be rapidly outcompeted by residents of the previous climax community that persisted through the disturbance. After a brief period of intermediate succession, a new climax community will form and persist until another perturbation occurs and the cycle of secondary succession begins anew. This process of ecological succession is a mechanism that could describe the cyclic nature of PEs in CF patients and the natural progression of lung disease.

## ECOLOGY-INFLUENCED APPROACHES TO TREAT CF LUNG INFECTIONS

As we continue to search for novel strategies to treat and manage chronic infections in individuals with CF, there may be advantages to identifying treatments that take ecological and evolutionary principles into consideration. Many authors have suggested that the manipulation of CF microbial communities, both in terms of taxonomic composition and metabolic function, could be a suitable alternative to traditional antibiotic therapies (8, 146). These could be conceived in the form of: (i) increased microbial diversity via supplementation, (ii) alteration of microbial metabolic networks, or (iii) targeted physiological changes within the lung microenvironment.

The notion that milder disease is associated with increased community diversity may indicate that boosting its structure and diversity by artificial means may be beneficial (16). For example, dietary supplementation with Lactobacillus casei has been shown to stimulate alveolar macrophages in mice, leading to enhanced clearance of respiratory *P. aeruginosa* (147). A similar approach that could increase microbial diversity within the lung or drive the community toward that of an earlier succession state may be beneficial.

While there is significant interpatient variation in CF microbial community composition, the functional microbial metagenome is less variable (31). This could provide an opportunity to target conserved functional pathways among common organisms. The suppression of a keystone species such as *P. aeruginosa* in this manner may initiate a cascade that negatively affects interspecies associations, biofilm formation, metabolic cross-feeding and antibiotic resistance, thereby altering pathogen virulence and the dynamics of infection. Similarly, disrupting essential functional pathways such as the nonmevalonate pathway of *Rothia* spp. or amino acid metabolism may help to reduce the overall microbial burden in the airways; however, targeting lung populations exclusively without altering other extrapulmonary microbiota may prove difficult (28).

Another element of ecological theory that could be applied toward CF treatment is that interspecies interactions may be either agonistic or antagonistic in different situations. Interactions can occur both within the same level of the trophic web (i.e., between bacteria) or between levels of the trophic web (i.e., between bacteria and host). The impact of these interactions can manifest in terms of net effects on the ecosystem and environment, or in the case of CF, the patient. As discussed previously, some anaerobe-mediated actions, such as cross-feeding of SCFAs, can enhance the growth of traditional CF pathogens (148), whereas others, such as changes in pH, negatively impact the viability of traditional pathogens (44). Enhancing the antagonistic interactions between bacteria, in particular stimulating pH changes, may limit the pathogenicity of specific CF communities. In contrast, disrupting interactions between species, such as those between *P. aeruginosa* and filamentous bacteriophages, may also be useful. Preventing these interactions may limit the development of phenotypes that enhance pathogen fitness in CF (e.g., biofilm formation and antibiotic tolerance) since many phage-infected strains exhibit these properties and are often associated with worsening lung function during PEs (149).

## CONCLUSIONS AND FUTURE DIRECTIONS

Life expectancy for CF patients has risen significantly since the initial description of the disease, from death during infancy being common in the 1950s to a current median age of survival of more than 50 years in North America (150, 151). However, chronic polymicrobial lung infections remain the largest cause of morbidity and mortality, and therefore the greatest barrier to improving clinical outcomes (152). The cystic fibrosis ecological succession (CFES) model synthesizes the dynamics of CF lower airways communities over the course of disease using fundamental concepts of ecology. Each stage of ecological succession within the CF microbiota is associated with specific clinically observable pathologies and contributes to the progressive lung decline and, ultimately, premature death. The CFES model aims to enhance existing ecological models of CF infections with a longitudinal perspective that describes CF airway ecology, while highlighting the species and metabolic mechanisms involved in progression of CF lung disease.

A more complete understanding of the CF lung ecosystem requires additional longitudinal studies of the microbiome, metagenome, metatranscriptome, and metabolome to fully characterize the dynamics and diversity of this system. Long-term longitudinal studies of ecological succession in the CF lung will help separate out correlation from causation and identify host and microbiological factors that trigger or facilitate PEs, therapeutic failure, and disease development. A limitation of all current CF ecological models, including the CFES described here, is our poor understanding of the host’s role in driving CF lung ecology, including local immunogenic, structural, and physiochemical changes that result in the provision of nutrients, substrates, or antimicrobial compounds or conditions (153).

While it is well established that traditional CF pathogens play key roles in CF infections, a growing literature indicates that there is also an important role for unconventional CF taxa like anaerobic species in disease pathology. Significant discourse still exists in the literature surrounding the role of unconventional pathogens, the mechanisms of PEs, and other elements of CF disease pathology. Due to variations in the microbiotas of individual patients and patient cohorts between studies, it is difficult to know whether seemingly contradicting results are truly discordant or simply reflect the diversity of CF microbial infections or sampling strategies. There has been interest in including viruses and fungi into ecological models of CF due to their presence in culture-independent data sets and association with clinically significant events such as PEs (15, 154–160). However, the role of these microbes in CF lung disease is not well understood and requires further study.

As explored in this review, there is immense spatial and temporal variation in the CF microbiome, both within and between patients. With the advent of personalized medicine, the profiling of patient-specific microbial communities will become increasing important in developing effective treatments that aim to match the dynamic nature of CF infection. This will require not only better clinically accessible methods that can provide robust species- and strain-level identification of microbes but also a comprehensive model of CF microbial ecology, which we have proposed here. The use of these ecological principles may be able to allow for improvements to CF treatment and provide a solution to contemporary challenges in effective disease management such as pulmonary exacerbations and antimicrobial resistance.
